# Balint groups’ possible role in self-care and job satisfaction of general practitioners—A qualitative study

**DOI:** 10.1007/s00508-024-02404-7

**Published:** 2024-08-06

**Authors:** Karina Schweiger, Benedikt Hofbaur, Susanne Rabady

**Affiliations:** 1 Karl Landsteiner University of Health Sciences, Dr. Karl-Dorrek-Straße 30, 3500 Krems, Austria; 2 Division General and Family Medicine, Department of General Health Studies, Karl Landsteiner University of Health Sciences, Dr. Karl-Dorrek-Straße 30, 3500 Krems, Austria; 3 Division General and Family Medicine, Department of General Health Studies, Karl Landsteiner University of Health Sciences, Dr. Karl-Dorrek-Straße 30, 3500 Krems, Austria

**Keywords:** Balint groups, Primary care physician, Job satisfaction, Self-care, Burnout prophylaxis, Motivation

## Abstract

**Background:**

Concerns are growing when it comes to the shortage of primary care physicians, therefore it seems necessary to take a closer look at job satisfaction and self-care as one of many influences on career choice. A higher job satisfaction reduces the risk to experience burnout and job-related stress and in addition it will contribute to staying in the profession. The objective of this study is to investigate the impact of regular participation in Balint groups on job satisfaction and self-care among general practitioners.

**Methods:**

Descriptive qualitative study with semi-structured expert interviews of 7 general practitioners. Thematical analysis of data and narrative summary.

**Results:**

A total of 402 coded segments were categorized into 8 main themes and 39 subthemes. Interviewees emphasized changes in self-care and job satisfaction as a result of Balint work and mentioned Balint work as a burnout prophylaxis for themselves. Competences that were learnt or improved through Balint work were described as well as aspects such as feedback and connection with colleagues or professional challenges and difficulties.

**Conclusion:**

The results of the study give rise to the assumption that regular participation in Balint groups might help to improve self-care, resilience, and contribute to job satisfaction. Further research is needed before a general recommendation can be made. Many positive aspects were described by the experts, while no harmful negative influences of Balint work were identified.

## Background

In many countries around the world, there is growing concern about the shortage of primary care physicians. Steps are being taken to increase the number of primary care physicians but attracting young physicians to this career path remains a challenge [1]. Various factors determine physicians’ career choices or contribute to remaining in the profession. One of those factors is job satisfaction, which appears to play an important role [2–9]. This study concentrated on job satisfaction.

Job satisfaction in itself is influenced by many factors. A study of 7379 primary care physicians from mainly European countries showed that feedback from colleagues, patient satisfaction, comfort and behavioral reassurance are important in increasing job satisfaction [3]. Other studies have identified factors such as variety, relationships and contact with colleagues and teaching medical students [4]. In addition, job satisfaction is considered to be positively affected by broad medical knowledge with opportunities for continuing education and skill development. Higher job satisfaction can reduce job stress and the risk of burnout. To increase job satisfaction, specific skills in managing care and communicating with patients are key requirements [2]. A patient-centered approach with a high-quality patient-physician relationship and appropriate self-care is essential [5]. The physician’s well-being and job satisfaction is also important for patients. It can influence the physician-patient relationship and patient satisfaction [6]. These aspects indicate the need to address methods to improve job satisfaction and self-care.

We wanted to know if self-care and guided reflection as offered by Balint work can contribute to job satisfaction in the field of general medicine. Therefore, this study focused on Balint groups and their possible effect. Balint groups were developed by Michael and Enid Balint and they led the first Balint group in the 1950s [11]. Groups of 8–12 physicians and a psychotherapist as group leader, hold regular case conferences lasting at least 2h. This group work aims to improve the awareness of psychological factors in patient care and enables the general practitioner (GP) to gain a deeper understanding of the patient’s problems and diseases [12]. In the long term, the physician’s inner attitude, approach and behavior may change, which is often recognized by the patient [13]. Some psychosomatic basic care courses include Balint work as a mandatory training. These mandatory courses were evaluated in Germany using feedback forms by 1667 doctors participating in a 10-unit course between 2004 and 2019. The long-term effects of Balint group participation were not considered, but the study resulted in good to very good ratings in terms of knowledge gain and positive impact on everyday medical practice [14]. The studies and numbers of probands are limited, but they do suggest that Balint work could have a positive impact on the GP’s job satisfaction [10].

The patient-centered approach could assist in aligning ethical and personal expectations of GPs with reality and thus increase job satisfaction and reduce the risk of burnout [15]. A cross-sectional study from Belgrade, involving 210 physicians also concluded that participating in Balint groups reduces the level of burnout compared to physicians not participating in Balint groups [16]. A qualitative study from Sweden, which conducted focus group interviews with 19 members of 4 different Balint groups, showed that stress relief and anxiety reduction were important effects. Participants mentioned a positive influence on resilience, a changed perspective on work and a better quality of life [17]. Little attention has been paid to job satisfaction so far, therefore the aim of this study was to investigate the effects of regular participation in Balint groups on the job satisfaction and self-care of GPs through expert interviews.

## Methods

### Study design

The study design is a descriptive qualitative study. Semi-structured expert interviews were conducted with GPs participating in a Balint group (Table 3). A literature review was carried out to obtain information on the interview guide. The study was conducted according to the consolidated criteria for reporting qualitative research (COREQ) (Table 4; [18]).

### Sampling strategy

Criteria for the selection of participants were established prior to the start of the recruitment process: Participants should have worked as GPs for at least 3 years and attended Balint groups regularly for at least 1 year. These criteria were chosen to gain a broad insight into the job satisfaction and self-care and to be able to assess the long-term influences of Balint work. No more than two participants should be recruited from the same Balint group, to ensure a wider range of experiences. The selection of participants was purposive. Through the Austrian Balint Society, Balint group leaders in Austria were contacted to recruit participants for the expert interviews. To avoid bias, the group leaders were not informed about the exact topic of the study or the research question and 5 Balint group leaders passed on the contact details of the interviewer to the participants of their groups. To ensure voluntary participation, participants contacted the interviewer personally by e‑mail or telephone. During the initial contact with the participants, no information about the content of the interview was given. The participants were unknown to the interviewer prior to the interview, they were only informed that the interviewer was a medical student and 7 participants agreed to the interview after the first contact. There were no dropouts.

### Data collection

One-to-one semi-structured expert interviews were conducted. Participants were asked to find a convenient time that did not interfere with their routine business and allowed privacy. Out of 7 interviews 6 took place online via “MS Teams” or “Zoom”, while one interview was conducted face to face. The interviews were audio and video recorded, Additional information and specifics of the interview guide were noted in handwritten form. The interviews lasted on average 20–25 min, not including the introduction and conclusion. The transcripts were anonymized using participant numbers instead of names and specific details were generalized. The original records and transcripts are kept in a password-protected file. The interviews were conducted by the first author of this paper. The first author of this paper is a female medical student with 9 years of prior experience as a nurse.

### Research question

Can participation in Balint groups be recommended to improve self-care and job satisfaction among GPs?

### Data analysis

The interviews were transcribed and coded by the first author. The coding software “MAXQDA” was used with a thematic analysis according to Brown and Clarke [19]. Prior to coding, the main categories were deductively derived from the literature and the interview guide. After an initial round of deductive coding, specific statements were inductively coded and assigned to the main categories. The results of the data analysis were summarized in tabular form and each main theme was described narratively. Quotes from the participants were used to supplement the themes. The results were discussed and compared with the literature findings. For the linguistic and grammatical correction of the paper, “DeepL Write” was used.

### Ethical consideration and data protection

At the beginning of the interview, the participants were informed about the procedure and the purpose of the interview. The data collected were anonymized at the first possible stage, when the interviews were transcribed. For all interviews, a written informed consent form was signed by the participants. The project was conducted in accordance with the Declaration of Helsinki (1964) and all subsequent updates of the Declaration, with the European Commission’s “Guideline of Good Clinical Practice”, with national requirements and with the Karl Landsteiner University of Health Sciences in Krems. The ethics committee was contacted to confirm that ethics approval was not required as no patient or health-related data were collected, and expert data were anonymized.

## Results

The analysis of the interview transcripts resulted in 8 main themes and 39 subthemes. A main theme focuses on the description of the interviewees and 7 main themes focus on the experiences and influences of Balint work. Details are given in Table 1.

**Table 1:** Table of main themes and subthemes

Main themes	Subthemes
Description of interviewees	Professional experience in years
	Balint group participation in years
	Job satisfaction at the time of the interview
	Self-care at the time of the interview
	Professional challenges and difficulties
Competencies learned or improved through Balint work	Communication skills
	New perspectives
	Patient-physician relationship
	Feedback
	More composure
Changes in self-care through Balint work	Self-reflection and new perspectives
	Self-care and self-confidence
	Less perfectionism
	Motivation and orientation
Job satisfaction and Balint work	Better interaction with patients and oneself
	Career changes
	Changes in interaction with employees
	Empathy training
	Reflecting on routines
	Observation/nonverbal hints
	Self-reflection and setting boundaries
	Attitude change
	Patient-centered approach
	Conflict management
	Difficult patients
	Job satisfaction positively influenced
	Job satisfaction not directly influenced
Balint work as burnout prophylaxis	Perceived as burnout prophylaxis
	Not perceived as burnout prophylaxis
Feedback and connection with colleagues	Networking and exchange
	Learning from different approaches
	Different fields of expertise
	Openness in a protected space
Possible negative experiences	Costs, time required, travel distance
	Dealing with feedback or criticism
	No negative experiences
Recommendation of Balint work	Absolute recommendation
	Hospitals and institutions
	Continuing education and training

### Description of interviewees

There were 7 participants in the interview sample. The average professional experience was more than 18 years and the average length of participation in Balint groups was more than 10 years. Participants were recruited from 4 different Austrian provinces and from 6 different Balint groups. Details are given in Table 2.

**Table 2:** Demographics of the participants

Variable	n
All	7
*State*	
Lower Austria	2
Upper Austria	1
Salzburg	3
Vienna	1
*Sex*	
Female	6
Male	1
*Work experience* *(as primary care physician)*	
8 years	1
14–15 years	3
20 years	1
> 30 years	2
*Balint group participation*	
4–7 years	5
16 years	1
35 years	1

Of the participants 5 worked in the public sector and 2 participants worked in the private sector, 2 participants worked full-time in their own general practice from Monday to Friday (one in a large city in Austria, the other one in a more rural area), with contracts to all Austrian health insurances. Of the participants 2 worked in a group practice also with contracts to all Austrian health insurances, one of them full time and another 30 h per week with additional 10 h of private appointments. Another primary care physician working in the public sector works as a GP in a hospital and as school physician, 2 primary care physicians currently work in the private sector in their own elective general medical practice and one of them additionally works in a rehabilitation center. One of them has worked as a substitute primary care physician in the public sector for many years.

#### Job satisfaction at the time of the interview

Out of 7 experts 6 rated their job satisfaction as high to very high. The interviewees named the following aspects that contribute to a high level of job satisfaction: self-determined work, variety in everyday working life, daily reflection, collaboration and exchange with colleagues, opportunities for continuing education and training, working with students, being aware of one’s own needs, setting boundaries and taking breaks. The patient-physician relationship, successful patient contacts, patient-centered work, and the ability to take time for the patients were highlighted as particularly conducive to job satisfaction. One expert rated the job satisfaction as rather low. This is mainly due to structural and financial challenges.

#### Self-care of the interviewees at the time of the interview

One expert described self-care as very good, thanks to an ideal work-life balance and changes in the professional environment. Of the experts three described self-care as an ongoing process that has to be learned slowly and should be renewed and made conscious on a regular basis. While two experts described their self-care as in need of improvement, another expert stated that self-care is currently clearly neglected due to personal problems. Activities and ways to improve self-care mentioned by the experts include exercise, nature walks, gardening, spending time with loved ones, taking rest periods, getting enough sleep, listening to music, attending cultural events, and an overall healthy lifestyle. Experts highlighted time management and breaks between patient appointments as critical to providing quality care and mentioned that small rituals are a good way to calm down during the working day.

#### Professional challenges and difficulties

The experts described some important challenges and difficulties in their professional life. Time seems to be a major challenge in everyday work. Busy work schedules leave little time for breaks or to address conflicts and problems, which can have a negative impact on the patient-physician relationship, quality of care and satisfaction for both parties. Pressure situations make it difficult to attend to personal needs.

“If I have to see 100 patients in a day, I can’t deal with conflicts and problems the way I want to, and things stay open. If I have to fill in an insane amount of paperwork and have no time at all to deal with the person, these are the systemic factors that have a huge impact on the patient-physician relationship, on the quality of care, and also on patient and physician satisfaction.” (Interview 6)

Another challenge, described by some experts, is that primary care physicians work alone most of the time. The workload cannot be shared, and decisions are always made by one person alone. One expert described working alone as an advantage as it leads to self-determination.

### Competencies learned or improved through Balint work

Some experts experienced an improvement in their communication skills, especially when it comes to engaging with patients and taking their needs seriously. One expert summarized this as “empathy training”. Another expert saw a change in the own view of the patient-physician relationship as a result of participating in Balint groups.

“It helps to listen in a different way and to take the patient’s needs very seriously, which I didn’t manage to do before. Now I can definitely engage better with patients.” (Interview 7)

In the opinion of the experts, the acceptance of positive feedback and appreciation is also trained in Balint group discussions. It teaches how to give constructive feedback and how to motivate colleagues. This increases self-confidence and thus influences the feeling of security. Broadening horizons, thinking from different perspectives and implementing new ideas and job opportunities are also mentioned as effects of Balint group participation. One expert described Balint work as “training to calm down”.

“I think sometimes it is not bad to have a different point of view, to learn to think from a different position and just to be more open.” (Interview 4)

“I would consider more composure as an absolute acquisition of competence.” (Interview 1)

### Changes in self-care through Balint work

Balint work improved the self-care of the experts in several ways. In summary, experts stated that it leads to a more loving and reflective approach to oneself and reduces pressure or perfectionism. It trains dealing with mistakes and self-forgiveness, setting boundaries, and taking breaks. A positive impact on self-appreciation and self-reflection was mentioned. For some experts this led to an increase in empathy and sensitivity towards patients and towards oneself. One expert pointed out that the Balint group could also be used to discuss personal or private conflicts and stresses, which also has an impact on self-care.

“It also trains my introspection in terms of self-care: When do I need a break? When do I need a moment on the terrace to take a deep breath? How do I feel after a touching or difficult conversation? It trains me to take care of myself a lot.” (Interview 3)

### Job satisfaction and Balint work

Of the experts four see a definite positive influence on job satisfaction, one of them even a massive influence. A fifth expert cannot attribute the improved job satisfaction solely to Balint groups due to additional training and methods used but sees a large part in it. Another expert sees no direct effect on job satisfaction, according to this expert job satisfaction is high anyway. In contrast, the expert with the lowest job satisfaction does not see an influence of Balint group participation on job satisfaction, as working with patients is only part of the job and the main problems of dissatisfaction are more structural and financial. Nevertheless, the expert describes that a few days after the Balint group, work feels more relaxed and an open attitude improves. Overall, 5 out of 7 experts see a definite improvement in job satisfaction through regular participation in Balint groups, while 2 experts do not see job satisfaction itself directly influenced.

According to some experts, Balint work can change the way they care for patients and allows them to question one’s own attitude. It helps to analyze challenging situations, to reduce operational blindness and to understand personal triggers and issues. This self-reflection allows for a professional distance from the patient while still allowing for the expression of emotions. One expert stated that understanding personal nuances and issues can lead to better patient care and even prevent burnout.

“It really helped to gain security or to resolve conflicts, to see problems in a more differentiated way, to find a way to deal with patients where it might have been difficult.” (Interview 6)

One expert recalled that Balint work had led to positive developments in dealing with employees. Empathy towards colleagues and employees had changed in some cases, and it was described that one’s own frustration tolerance had become greater.

“There is a greater sense of togetherness. My own empathy towards colleagues has also changed.” (Interview 2)

Another aspect mentioned is that Balint work can help to continually reflect on personality traits and needs. In the case of one expert, the result was an increase in joy and love of the medical profession. In some cases, Balint work has led to changes in the career path, such as specialization in a particular medical field, a change in working environment, or an increased focus on psychosocial and psychosomatic approaches.

“By feeling this different quality of relationships, which can be worked out so finely in Balint groups, it has actually had a direct influence on my career.” (Interview 6)

Of the experts four addressed the issue of “difficult patients”. Case discussions and feedback from colleagues help to promote a sense of calm and approach patients with less emotional attachment and more curiosity. This can reduce the burden of difficult cases and prevent some patients from being negatively labelled when they appear on the waiting list.

“There are no more irritating or negative feelings when looking at the waiting list. Everyone is called in the same way, with the same practiced calmness.” (Interview 1)

### Balint work as burnout prophylaxis

Out of 7 experts 6 clearly consider Balint work as a burnout prophylaxis for themselves. One expert does not see it as a burnout prophylaxis, as other methods seem to be more suitable in this case; however, according to this expert, Balint groups seem to be a good tool to reflect on job satisfaction and to become aware of personal and professional developments.

For one expert Balint group participation has a relieving effect and leads to fewer professional worries to take home, which results in a better end of the working day and increases the ability to relax. Other experts described a positive impact on factors such as handling difficult or overwhelming situations, recognizing blind spots, finding easier ways of doing things or reducing the pressure to perform. One expert sees Balint groups as the “best burnout prophylaxes available” and highlighted social togetherness, solidarity and “being seen” as positive factors.

“You know that you can put these worries elsewhere and work on them in the next Balint group session.” (Interview 3)

### Feedback and connection with colleagues

Experts agreed that Balint work provides a valuable opportunity for personal and emotional exchange and a safe and confidential space for honest and open communication. The diversity of specialties in the group enables cross-disciplinary thinking and learning, which is seen as a positive development by experts. Discussing difficult patient cases and sharing experiences can improve the quality of care and normalize the experience of making mistakes and facing challenges in healthcare. The connection with colleagues was generally emphasized as a great motivating factor for regular participation in Balint groups.

“It is also pleasant to be in such a group. You feel that it is a special atmosphere or environment.” (Interview 2)

### Possible negative experiences

It was mentioned that it is sometimes difficult to fit the sessions into the daily work routine or to motivate oneself after a long working day but that it is still time well spent. It was also noted that the travelling distance is very long for some. One expert pointed out, that parking fees are high because the Balint group meets in the city center.

When asked if individual Balint group sessions had a negative impact on job satisfaction, 7 out of 7 answered “no”; however, some experts pointed out aspects considered less ideal. One expert said that although it does not negatively influence job satisfaction, it sometimes has an effect on personal satisfaction because it brings situations to the surface that could have been handled better. Indirect criticism, feedback or opinions that are not in line with one’s own can lead to a temporary reduction of self-esteem but this also encourages self-reflection, according to this expert. Another expert stated that one has to be able to deal with harsher attacks or criticism if sessions do not proceed professionally. A too large or too small group size was also mentioned as less ideal.

### Recommendation of Balint work

Experts concluded that Balint work should be recommended to increase self-care and job satisfaction, not only for physicians but also for other health professions and it should be included in the education as well. It should not be mandatory but should be strongly encouraged and presented in a way that is more likely to be accepted.

“I can recommend it to any physician, whether in a hospital setting or in outpatient care. I know it is only meant for physicians, but in reality any person who deals with other people would need it.” (Interview 3)

## Discussion

Our interview partners very homogeneously showed relatively high job satisfaction. Their own perception made a clear connection to their longstanding participation in Balint groups. In spite of the relatively small number of participants, we formed a first hypothesis that regular participation in Balint groups may have a positive impact on job satisfaction. It appears to increase resilience and helped to accept structural or financial factors that cannot be easily changed.

This hypothesis is considered to be strengthened by the fact that participants are working in a broad variety of settings. What they all share, except for being GPs, is Balint work over a longer period of at least 1 year.

Our results are in line with studies summarized in the background in terms of job satisfaction, burnout prevention and the professional identity [15–17]. Factors such as the training of a patient-centered view, a better handling of difficult patients, dealing with insecurity and mistakes, or an increased empathy towards colleagues [15] were mentioned by the experts as well. It should, in addition, be mentioned that the interviewees in this study also largely see participation in Balint groups as burnout prevention.

We consider our results to be relevant triggers for further research into a most important topic, including a comparison with non-participants or dropouts. Exploring a wider range of Balint groups, particularly in clinical settings and in different specialties, would be interesting to understand the impact on the work climate and job satisfaction of physicians in the outpatient and inpatient sector.

## Strengths and limitations of the study

A clear limitation of the study is the small sample size, although there was data saturation on the main themes and the experts show a very long experience with Balint groups compared to other studies.

Interviewer bias can be a potential problem. The interviewer’s expectations and personal opinions can influence the atmosphere and direction of the interview. To reduce bias, an interview guide was prepared and open-ended questions were asked. Leading questions were avoided altogether and questions for clarification and control were asked. The interviewer did not participate in a Balint group more than five times in order to remain as unbiased as possible and to analyze the method from an outside perspective. This is a strength compared to studies conducted by Balint group leaders or long-term Balint group participants.

Volunteer bias should be mentioned as well. It is possible that physicians who are more satisfied are more inclined to participate in an interview than those who are generally more overworked or stressed. To reduce this, the participants were not informed about the topic of the interview. They did not know in advance that it would focus on job satisfaction and self-care.

Nevertheless, the homogeneity of the results creates interest for further research. As current research on this specific research question is limited, the comparability of the results with other studies is only possible to a certain extent.

We did not study the influence of Balint groups on coping with the particular strain working in the public sector (“primary care”) might imply. This aspect would also merit particular attention in further research.

## Conclusion

The results of the study suggest that regular participation in Balint groups may help to improve self-care and job satisfaction in GPs. No harmful effects could be identified. Further research with larger samples, and a comparison with physicians not taking part or having dropped out of Balint groups is needed to assess the replicability of these findings on a wider scale, before general recommendations can be made.

## Data Availability

All data are stored in a password-protected file on the server of the first author. All data were anonymized before the analysis.

## Appendix

**Table 3:** Interview guide

Interview guide	Category
Introduction/general Information/data use	Introduction
Since when and how often do you participate in Balint groups?	Participant criteria
How long have you been working as a general practitioner?	
How would you currently describe your satisfaction as a general practitioner?	General satisfaction
What helps you to promote your own satisfaction in the profession?	
How would you describe the way you deal with yourself? (keyword: self-care)	
How did you become aware of Balint groups and why did you start participating?	Balint groups: general questions
What motivates you to regularly participate in Balint groups?	
What competencies were you able to acquire/improve by participating in Balint groups?	
To what extent does participation in Balint groups change the way you treat yourself?	Self-care/job-satisfaction
How has participation in Balint groups influenced/changed your behavior at work?	
Would you say that your job satisfaction is influenced by participation in Balint groups and can you describe this?	
Does participation in Balint groups serve as burnout prophylaxis for you? (Additional question: If yes, to what extent? If no, what else works for you?)	
Would you recommend participation in Balint groups to colleagues to improve job satisfaction and self-care?	
Are there any negative experiences/disadvantages for you as a result of participating in Balint groups?	Control questions
Have single Balint group sessions adversely affected your job satisfaction?	
Finally, is there anything else you would like to say about Balint groups?	Completion of the interview

**Table 4:** Consolidated criteria for reporting qualitative studies (COREQ): 32-item checklist [18]

No. item	Guide questions/description	Reported on
**Domain 1: Research team and reflexivity**		
*Personal characteristics*		
1. Interviewer/facilitator	Which author conducted the interview or focus group?	Material and methods, data collection
2. Credentials	What were the researcher’s credentials?	Material and methods, data collection
3. Occupation	What was their occupation at the time of the study?	Material and methods, data collection
4. Gender	Was the researcher male or female?	Material and methods, data collection
5. Experience and training	What experience or training did the researcher have?	Material and methods, data collection
*Relationship with participants*		
6. Relationship established	Was a relationship established prior to the study commencement?	Material and methods, sampling strategy
7. Participant knowledge of the interviewer	What did the participants know about the researcher?	Material and methods, sampling strategy
8. Interviewer characteristics	What characteristics were reported about the interviewer?	Material and methods, sampling strategy
**Domain 2: Study design**		
*Theoretical framework*		
9. Methodological orientation and theory	What methodological orientation was stated to underpin the study?	Material and methods, study design
*Participant selection*		
10. Sampling	How were participants selected?	Material and methods, sampling strategy
11. Method of approach	How were participants approached?	Material and methods, sampling strategy
12. Sample size	How many participants were in the study?	Material and methods, sampling strategy & results
13. Non-participation	How many people refused to participate or dropped out?	Material and methods, sampling strategy
*Setting*		
14. Setting of data collection	Where was the data collected?	Material and methods, data collection
15. Presence of non-participants	Was anyone else present besides the participants and researchers?	Material and methods, data collection
16. Description of sample	What are the important characteristics of the sample?	Results, description of interviewees
*Data collection*		
17. Interview guide	Were questions, prompts, guides provided by the authors? Was it pilot tested?	Appendix
18. Repeat interviews	Were repeat interviews carried out? If yes, how many?	No
19. Audio/visual recording	Did the research use audio or visual recording to collect the data?	Material and methods, data collection
20. Field notes	Were filed notes made during and/or after the interview or focus group?	Material and methods, data collection
21. Duration	What was the duration of the interviews or focus groups?	Material and methods, data collection
22. Data saturation	Was data saturation discussed?	Discussion, limitations
23. Transcripts returned	Were transcripts returned to participants for comment and/or correction?	No
**Domain 3: Analysis and findings**		
*Data analysis*		
24. Number of data coders	How many data coders coded the data?	Material and methods, data analysis
25. Description of coding tree	Did authors provide a description of the coding tree?	Material and methods, data analysis
26. Derivation of themes	Were themes identified in advanced or derived from the data?	Material and methods, data analysis
27. Software	What software, if applicable, was used to manage the data?	Material and methods, data analysis
28. Participant checking	Did participants provide feedback to the findings?	No
*Reporting*		
29. Quotations present	Were participant quotations presented to illustrate the themes/findings? Was each quotation identified?	Results
30. Data and findings consistent	Was there consistency between the data presented and the findings?	Results, discussion
31. Clarity of major themes	Were major themes clearly presented in the findings?	Results
32. Clarity of minor themes	Is there a description of diverse cases or discussion of minor themes?	Results
